# Suits for Alleged Malpractice

**Published:** 1888-05

**Authors:** 


### SUITS FOR ALLEGED MALPRACTICE.

Numerous suits for alleged malpractice have recently been entered in this and other cities against some of its eminent surgeons. The defendants are generally known to be careful and conscientious surgeons. Attention is called to the charge made simply to emphasize what is known to all, that character, above reproach in every way, does not protect its possessor from malicious prosecution. If human nature were free from venality, the ingratitude which prompts such suits might be allowed to pass without special comment, but there are believed to be, unfortunately, members of another learned profession always willing to undertake such prosecutions as a speculation.

Suits for alleged malpractice are susceptible of a dual division. In one are included all those in which the plaintiff really thinks the defendant guilty of the charge, when in point of fact he is not; and the other includes those in which some venal attorney makes his fee contingent upon his success in securing a verdict for the plaintiff, and urgently advises the bringing of the action at law. A case was recently decided in the courts at Boston in a manner which is interesting in this connection. The case was tried four times.

At the opening of the last trial a motion was made by the defense, and granted by the court, that in case the jury decided in favor of the defendant the plaintiff must pay the costs for all four of the trials. The verdict was for the defendant. This is just, as far as it goes, but is not sufficient.

The plaintiff bringing any such suit ought not only to pay all costs of unsuccessful trials, but ought also to be compelled by the law to give a bond for an amount sufficient to indemnify the defendant in case of failure of the plaintiff to establish the claim made. If unsuccessful, he should be compelled at least to pay, in addition to the costs of the trial, the expense in which the suit involved the defendant. This matter is one of interest to all physicians, since, as the instances cited show, no physician or surgeon is certain of immunity from such prosecution, and while he is not hedged about by a divinity which should protect him from criminal neglect of his duty, he and all other men should be guarded, more securely than at present, against the annoyance, injury to reputation, and the expense of malicious prosecution.

